# Response to Comments on “Poisonings with Suicidal Intent Aged 0–21 Years Reported to Poison Centers 2003–12”

**DOI:** 10.5811/westjem.2015.12.29429

**Published:** 2016-01-21

**Authors:** Sophia Sheikh

**Affiliations:** University of Florida College of Medicine-Jacksonville, Department of Emergency Medicine, Jacksonville, Florida

In Reply:

We chose to perform a sub-analysis on patients younger than age 10 as we were surprised to see such a relatively large number of intentional self-harming behaviors performed by patients in this age group. Given the paucity of published data for this age group we thought it important to characterize and discuss this particularly vulnerable population.

Details for the cases resulting in death, such as the exact ages, names of the 52 substances ingested, clinical effects, and therapies used, were analyzed but not included due to space limitations as this manuscript was submitted as a brief report. For those interested, the results are provided below (Tables 1–4).

Several studies, performed in the U.S. and internationally, have shown a relationship between ADHD and self-harm in the pediatric population.^1^,^2^ Impey M. performed a review of the literature and concluded based on the findings of 25 studies that patients with ADHD were more likely to have suicidal ideas and commit suicide attempts. This was true across all age groups (including the pediatric population).^3^ Our findings in conjunction with the literature brought us to suggest screening for ADHD in addition to mental health disorders in those patients presenting with self-harming behavior.

## Figures and Tables

**Table 1:** Deaths by reported age.

Age (years)	Number of patients
14	1
16	1
17	2
18	3
19	5
20	2
21	3

**Table 2:** Substances reported in death cases.

Substance	Cases
Unknown	15
Aspirin	4
Tylenol 500mg	3
Benadryl or diphenhydramine	2
Motrin 800mg	2
Seroquel	2
Adderall 20mg tablet	1
Amitriptyline 10mg	1
Anafranil 25mg	1
Benicar 5mg	1
Cartia xt 240mg	1
Depakote 250mg	1
Effexor 75mg	1
Euthanasia solution, T-61	1
Flecainide	1
Flexeril 10mg	1
Geodon	1
Ibuprofen	1
Lamictal 150mg	1
Lexapro 10mg	1
Methadone	1
Methamphetamine	1
Opiates	1
Paxil 40mg	1
Potassium	1
Pseudoephedrine	1
Trazadone	1
Strattera 18mg	1
Wellbutrin XL 150mg	1
Vicodin	1
Xanax	1

**Table 3:** Therapies used in death cases.

Therapies	Cases
Oxygen	15
Ventilator	13
Intubation	13
IVF	12
Alkalinization	10
Vasopressors	6
CPR	5
Atropine	4
Benzodiazepines	4
Charcoal, single-dose	4
Naloxone	3
NAC	3
Glucagon	2
Antiarrhythmics	2
Hemodialysis	2
Whole-bowel irrigation	2
Insulin	1
Flumazenil	1
Calcium	1

*IVF,* intravenous fluids; *CPR,* cardiopulmonary resuscitation; *NAC,* N-acetyl cysteine

**Table 4:** Clinical effects reported in death cases.

Clinical effects	Cases
Cardiac arrest	12
Asystole	11
Respiratory arrest	10
Coma	9
Tachycardia	8
Hypotension	7
Vomiting	5
Hyperventilation/tachypnea	4
Conduction disturbance	3
Drowsiness/lethargy	3
Nausea	3
Hyperglycemia	3
Acidosis	3
Respiratory depression	3
ECG change (other)	2
Hypertension	2
Seizure (single)	2
Mydriasis	2
Tinnitus	2
Diaphoresis	2
Bradycardia	2
Confusion	1
X-ray findings (+)	1
Abdominal pain	1
CPK elevated	1
Dysrhythmia (other)	1
AST, ALT >1000	1
Seizures (multiple, discreet)	1
Syncope	1
Chest pain (noncardiac)	1
Increased creatinine	1
Dizziness/vertigo	1
Diarrhea	1
Increased anion gap	1
Cyanosis	1

*ECG,* electrocardiogram; *CPK,* creatine phosphokinase; *AST,* aspartate aminotransferase; *ALT,* alanine aminotransferase

## References

[1] Restrepo-Bernal D, Bonfante-Olivares L, Torres de Galvis Y (2014). Suicidal behavior and attention decifit hyperactivity
disorder in adolescents of Medellin (Colombia),
2011–2012. Rev Colomb
Psiquiatr.

[2] Whalen DJ, Dixon-Gordon K, Belden AC (2015). Correlates and consequences of suicidal cognitions and
behaviors in children ages 3 to 7 years. J Am Acad Child
Adolesc
Psychiatry.

[3] Impey M, Heun R (2012). Completed suicide, ideation and attempt in attention
deficit hyperactivity disorder. Acta Psychiatr
Scand.

